# An Emerging Omega-3 Fatty Acid—Octadecapentaenoic Acid (18:5n-3): Biosynthesis, Potential Sources and Applications

**DOI:** 10.3390/md24100357

**Published:** 2026-10-12

**Authors:** Kaiwen Sun, Jamie Merilees, Xiao Qiu

**Affiliations:** Department of Food & Bioproduct Sciences, University of Saskatchewan, Saskatoon, SK S7N 5A8, Canada; kas731@usask.ca (K.S.); rse692@mail.usask.ca (J.M.)

**Keywords:** octadecapentaenoic acid (18:5n-3), omega-3 polyunsaturated fatty acids (PUFAs), microalgae, biosynthesis, biotechnological applications

## Abstract

Octadecapentaenoic acid (OPA, 18:5n-3) is an emerging and poorly characterized omega-3 polyunsaturated fatty acid (PUFA) that is primarily produced by certain marine microalgae. Although it is structurally similar to the well-studied eicosapentaenoic acid (EPA, 20:5n-3) and docosahexaenoic acid (DHA, 22:6n-3), OPA’s specific biological and ecological roles remain largely unknown. The purpose of this review is to comprehensively examine and critically analyze the current knowledge on OPA, including its biosynthesis, natural sources, and metabolic fate in consumers to assess its functional and nutraceutical potential. Current evidence suggests that OPA contributes to membrane structure in primary producers but is rapidly metabolized in consumers, acting more as an energy source or metabolic intermediate rather than a stored end product. While OPA shows the potential for applications in aquaculture and human nutrition, its practical utility is largely inferred from structurally related fatty acids. Many application questions remain unanswered due to its low natural abundance, analytical challenges, and a lack of targeted functional studies. Future research is therefore required for a better understanding its stability, bioactivity and metabolism to determine its true functional importance and practical uses.

## 1. Introduction

In recent years, omega-3 (ω-3) polyunsaturated fatty acids (PUFAs) have gained increasing attention due to their ability to promote general health and reduce the risk of many chronic diseases. These fatty acids are involved in numerous physiological processes, such as membrane structure, cellular signaling, inflammatory and immune activities, and brain, eye and cardiovascular functions [1,2,3,4]. Some of the most well-known ω-3 PUFAs include α-linolenic acid (ALA, 18:3n-3), eicosapentaenoic acid (EPA, 20:5n-3) and docosahexaenoic acid (DHA, 22:6n-3). ALA, primarily coming from plant sources, is an essential fatty acid for humans as they lack the ability to synthesize it themselves [5]. EPA and DHA are mainly derived from marine fish and heterotrophic microorganisms, and are considered the most biologically active members of the ω-3 group in humans and animals [6].

While EPA and DHA have been extensively studied for their health benefits and are predominantly applied as dietary supplements, there is growing interest in alternative and novel ω-3 fatty acids whose biological properties and health benefits are largely unexplored. One of these fatty acids is octadecapentaenoic acid (OPA, 18:5n-3), a highly unsaturated ω-3 PUFA produced only by certain microalgae and phytoplankton [7,8,9]. Even though OPA is structurally similar to EPA and DHA, it remains poorly characterized with its biosynthesis pathways, biological functions and potential applications. Because of OPA’s low natural abundance and certain analytical challenges, research has been limited, leaving many questions about its biosynthesis, metabolism, physiological roles and potential benefits unanswered. The aim of this review is to provide a comprehensive overview of OPA’s physicochemical properties, sources, biosynthesis, metabolism and biological function, and evaluate its potential applications in health and nutrition.

## 2. Chemical Structure and Physicochemical Properties of OPA

**Figure 1:**
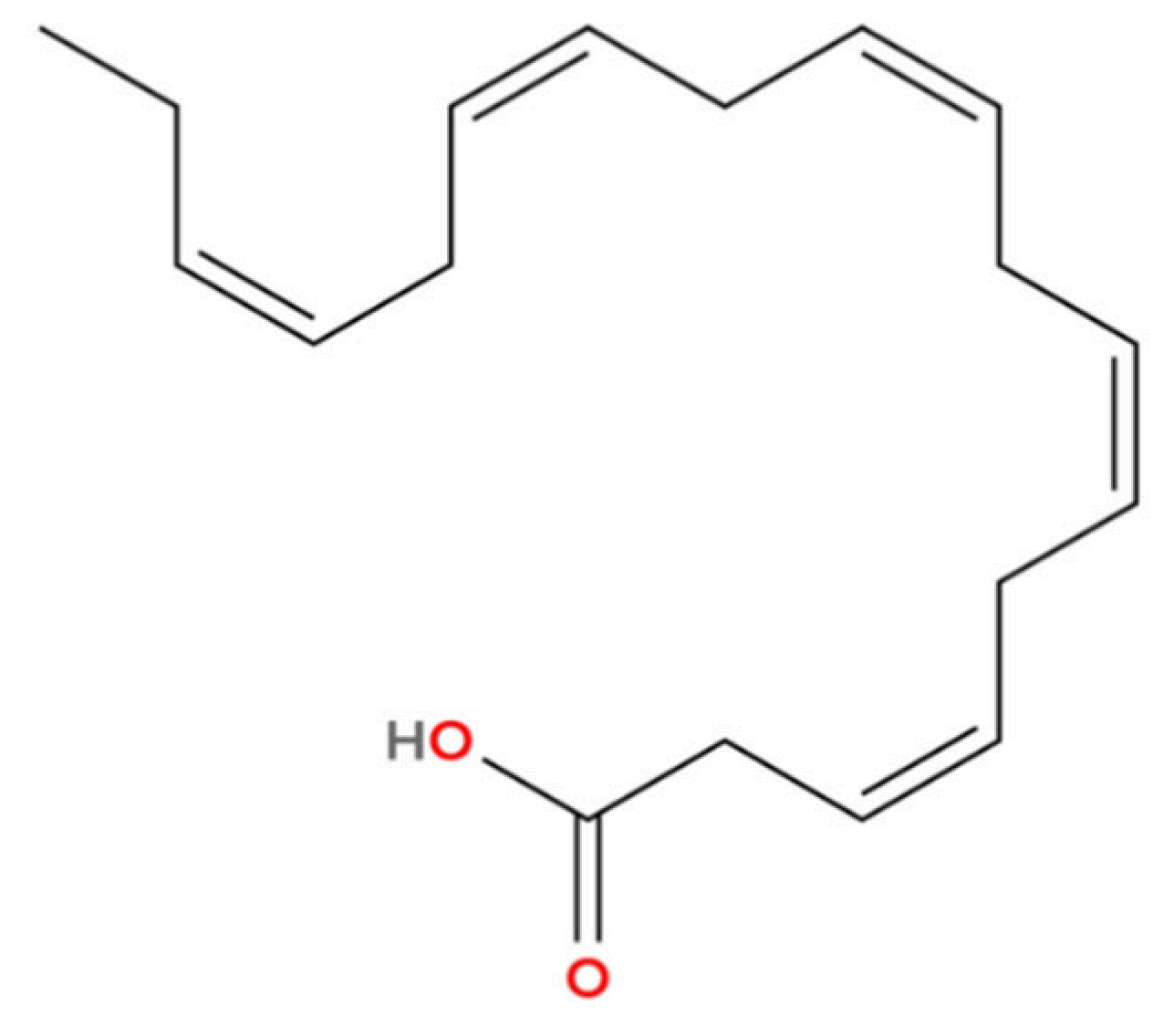
Chemical structure of octadecapentaenoic acid (18:5n-3). Created using MolView (version 2026-09-07, build d0731d4b; https://app.molview.com).

OPA is a highly polyunsaturated fatty acid (PUFA) with 18 carbons in chain length and five double bonds. All double bonds are in *cis* configuration, giving the molecule a strongly kinked structure (Figure 1). The first double bond from the methyl end is located at the third carbon, putting it in the ω-3 PUFA family. OPA can thus be notated as 18:5n-3. On the other hand, the first double bond from the carboxyl end is also located at the third carbon, putting it in the Δ3 unsaturated fatty acid. As such, OPA can also be denoted as 18:5-Δ3,Δ6,Δ9,Δ12,Δ15 or simply 18:5-3,6,9,12,15. Understanding the chemical nature is important, as it determines reactivity, biological function and potential applications.

The possible functional properties of OPA can be better understood through comparison with structurally related polyunsaturated fatty acids, as its biological properties are largely dictated by chain length, degree of unsaturation, and double bond position (Table 1). 

Compared to its direct precursor stearidonic acid (SDA, 18:4n-3), OPA contains an additional double bond from the carboxyl end (at the Δ3 position). This extra unsaturation distinguishes OPA from other 18 carbon chain ω-3 PUFAs, such as SDA and α-linolenic acid (ALA, 18:3n-3), that serve as metabolic precursors for ω-3 very long chain PUFAs (VLCPUFAs) [5]. Compared to major ω-3 VLCPUFAs such as EPA and DHA, OPA possesses a shorter carbon chain but a comparable or greater degree of unsaturation relative to its chain length. Specifically, EPA shares the same number of double bonds as OPA across a 20-carbon chain, while DHA contains one more double bond distributed along a 22-carbon chain. In contrast, arachidonic acid (ARA), a biologically important ω-6 PUFA, contains one fewer double bond than OPA within a 20-carbon chain [22]. These structural differences directly influence their incorporation into membrane lipids, oxidative stability and biological functions [1,25].

The high unsaturation of OPA brings notable physical properties. Increased chain bends brought about by five *cis* double bonds prevent OPA molecules from closely aligning with one another or with other fatty acyl chains, explaining its lower melting point and higher membrane fluidity [26]. This property directly influences membrane-associated processes, such as protein–lipid interactions and transmembrane transport [1].

A major trade-off of OPA’s high unsaturation is its susceptibility to oxidative degradation. In general, oxidation rates increase with degree of unsaturation; thus, OPA is significantly less stable than fatty acids with fewer double bonds. This high degree of unsaturation in OPA provides multiple sites for free radical attack, making it particularly susceptible to lipid peroxidation [25,27]. This oxidative instability represents a critical consideration for the handling, storage and practical application of OPA.

## 3. Biosynthesis and Metabolism of OPA

### 3.1. Biosynthesis of OPA

While OPA is recognized as a vital structural component of microalgal membrane lipids, its biosynthetic pathway has only recently begun to be elucidated. Eukaryotic microalgae introduce double bonds using membrane-bound fatty acid desaturase [28]. These desaturases are oxidoreductase that abstract two hydrogen atoms from a fatty acyl chain to form a carbon–carbon double bond, displaying strict substrate specificity and regioselectivity [29]. Unlike prokaryotes, which introduce double bonds during de novo fatty acid synthesis through the action of ketoacyl synthase and dehydratase, microalgal desaturases act sequentially on pre-formed fatty acyl chains.

In photosynthetic microalgae, de novo fatty acid biosynthesis originates in plastids where acetyl-CoA is converted to stearic acid (18:0) by a type II fatty acid synthase (FAS) [30]. Once synthesized, stearic acid can be desaturated to a monounsaturated fatty acid, such as oleic acid (OA, 18:1n-9) by a Δ9 desaturase. Oleic acid is then desaturated by a Δ12 desaturase to produce linoleic acid (LA, 18:2n-6), which can be further desaturated by a Δ15 desaturase to give ALA. Given that OPA contains five *cis* double bonds at the Δ3, Δ6, Δ9, Δ12 and Δ15 positions, its biosynthesis from saturated precursors requires five sequential desaturation steps, each requiring a specific desaturase [31,32,33].

**Figure 2:**
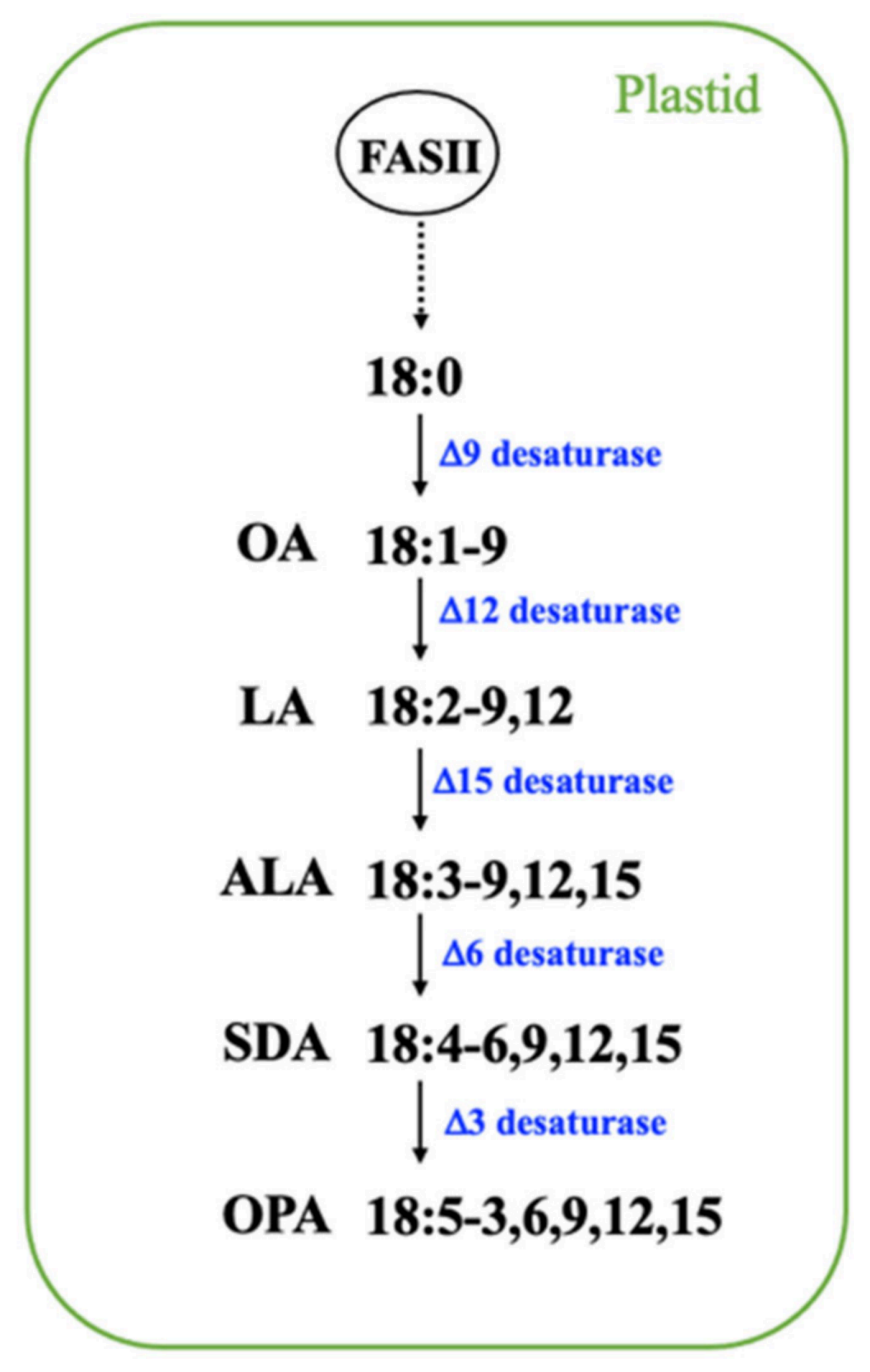
The biosynthetic pathway of OPA in microalga *Emiliania huxleyi*.

While these initial desaturation steps are well-established, how microalgae further synthesize the highly unsaturated OPA from ALA remained a long-standing question. This complete pathway was only recently mapped and confirmed in the haptophyte *Emiliania huxleyi*, a widely occurring eukaryotic unicellular marine microalga capable of producing a high level of OPA (23%), by our group using a series of radioisotope precursor-tracing experiments. By feeding *Emiliania* with ^14^C radiolabeled acetic acid and intermediate fatty acids, we provided the first biochemical proof that OPA is synthesized through sequential Δ12, Δ15, Δ6 and Δ3 desaturations occurring directly on plastidic galactolipids (Figure 2) [34].

Although trace amounts of OPA can be detected in phospholipid phosphatidylcholine (PC) or the thylakoid phospholipid phosphatidylglycerol (PG), OPA is preferentially enriched in the chloroplast envelope and thylakoid membrane galactolipids, predominantly monogalactosyldiacylglycerol (MGDG) and digalactosyldiacylglycerol (DGDG) [8,33,35,36]. For example, in the raphidophyte *Heterosigma akashiwo*, OPA accounted for 20.1% and 10.2% of the fatty acids in MGDG and DGDG, respectively, whereas only less than 0.3% was detected in the PC and sulfoquinovosyldiacylglycerol (SQDG) and PG fractions [37]. Similarly, in several dinoflagellates, including *Pyrocystis* spp., *Karenia brevis*, *Lepidodinium chlorophorum* and *Karenia mikimotoi*, OPA is a prominent constituent of MGDG and/or DGDG, consistent with its association with chloroplast and thylakoid membranes [38]. These observations, however, largely reflect the steady-state distribution of OPA among lipid classes and do not resolve the dynamic processes by which newly synthesized OPA is incorporated into specific glycerolipid pools. Recent radiochemical analysis in *E. huxleyi* provide further insight into how OPA is incorporated into glycerolipids. Using ^14^C-acetate and ^14^C-glycerol pulse-chase labeling, our group showed that OPA progressively accumulated in MGDG during the chase phase, concomitant with a decrease in its precursor OA and the appearance of intermediate desaturation products. OPA became the predominant newly synthesized PUFA in MGDG and was preferentially localized at the *sn*-1 position [39,40]. These results demonstrate that OPA is primarily synthesized through sequential desaturations at the *sn*-1 position of MGDG.

### 3.2. Metabolism of OPA

#### 3.2.1. Metabolic Remodeling of OPA Among Glycerolipids in Microalgae

Once incorporated into membrane lipids, OPA can undergo metabolic changes determining whether it is retained within glycerolipids, redistributed among lipid pools, oxidized, or released for further metabolism. Although OPA biosynthesis and its distribution among lipid classes have been examined, its metabolic change and subsequent fate remain largely unexplored. In microalgae, conditions such as nutrient deprivation, temperature acclimation and other stresses can induce extensive turnover of photosynthetic glycerolipids and the redistribution of fatty acids among membrane and storage lipid pools [39,40]. Because OPA is predominantly associated with MGDG and DGDG, such remodeling may alter its abundance and positional distribution within these galactolipids.

Our recent radiolabeling experiments in *E. huxleyi* provide preliminary evidence for positional remodeling of OPA within MGDG. Although newly synthesized OPA was initially detected predominantly at the *sn*-1 position, it subsequently accumulated at the sn-2 position during the chase phase, without a corresponding detection of OA at the *sn*-2 position [41]. This positional shift suggests that OPA may undergo acyl-chain remodeling within MGDG, potentially involving the exchange of OPA between the *sn*-1 and *sn*-2 positions, although the underlying enzymatic mechanism remains to be established. In addition, despite being incorporated primarily into galactolipids, OPA has also been detected in neutral storage lipids such as triacylglycerols (TAGs) in several species [32]. However, whether OPA is channeled into TAG directly through MGDG-derived DAG backbones or through alternative acyl-editing pathways remains to be fully explored.

#### 3.2.2. Metabolism of OPA in Higher Trophic Levels

Despite being synthesized in substantial amounts by marine microalgae [37,42], OPA rarely accumulates at higher trophic levels of living organisms. Because human and other mammals lack Δ12 and Δ15 desaturases required for the conversion of OA to LA, and LA ALA, they cannot synthesize ω-3 PUFAs de novo and must rely on the dietary intake of these essential fatty acids [43].

In humans and animals, dietary ALA can be metabolized to VLCPUFAs such as EPA and DHA through alternating desaturation and elongation reactions. This pathway converts ALA to SDA via an initial Δ6 desaturation, followed by Δ6 elongation to eicosatetraenoic acid (ETA, 20:4n-3), and Δ5 desaturation, giving EPA. EPA is subsequently elongated by a Δ5 elongase to produce docosapentaenoic acid (DPA, 22:5n-3). DHA biosynthesis then proceeds via the Sprecher pathway, involving further elongation of the DPA to tetracosapentaenoic acid (24:5n-3), Δ6 desaturation to tetracosahexaenoic acid (24:6n-3), and translocation to the peroxisome where one cycle of β-oxidation shortens the carbon chain by two carbons to yield DHA [44,45].

**Figure 3:**
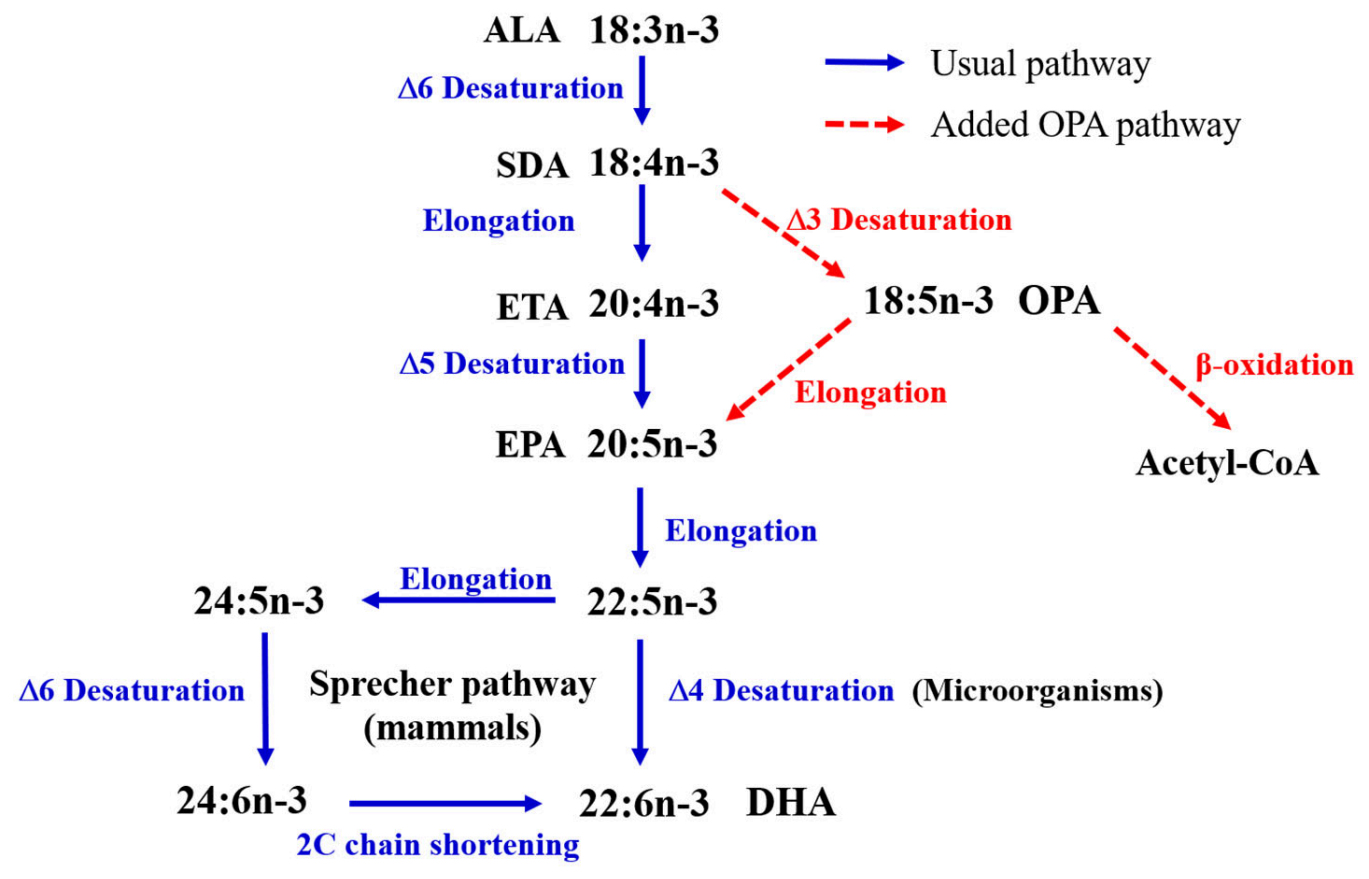
Possible metabolism of OPA in animals. The dash line denotes the added hypothetical OPA pathway for DHA biosynthesis in mammals. The Δ4 desaturation pathway for DHA biosynthesis occurs only in certain microorganisms.

However, this endogenous conversion is highly inefficient in mammals owing to rate-limiting enzymatic steps and competitive metabolic constraints; moreover, most ingested essential fatty acids are rapidly β-oxidized for energy generation [16]. For instance, in adult men, only 5–8% of ingested ALA is converted to EPA, and less than 0.5% is converted to DHA [15]. In this context, OPA, having already undergone more desaturations than ALA, may provide an alternative dietary intermediate for VLCPUFA biosynthesis. By having a pre-existing Δ6 double bond, OPA bypasses the rate-limiting Δ6 desaturation step required for the conversion of ALA to SDA, providing a potential substrate for EPA synthesis through a single elongation step (Figure 3). However, whether OPA is efficiently utilized through this pathway in animals has not been established.

In addition to its potential conversion to VLCPUFAs through elongation, OPA can also undergo β-oxidation-mediated metabolic reactions. In cultured fish cells, OPA was rapidly converted to SDA through a Δ3,Δ2-enoyl-CoA isomerase-mediated Δ3,Δ2-enoyl-CoA isomerase pathway. This initial β-oxidation-associated reaction removes the Δ3 double bond and generates SDA, which can subsequently undergo further metabolic processing, including elongation and desaturation toward very long-chain ω-3 PUFAs like EPA and DHA or further β-oxidation for energy production [7,17,46]. This rapid metabolic conversion may contribute to the generally low abundance of intact OPA at higher trophic levels. Indeed, the metabolic fate of dietary OPA in animals remains poorly understood. It is currently unclear as to what extent OPA is incorporated into tissue glycerolipids, converted to SDA and subsequently to longer-chain ω-3 PUFAs, or completely degraded via β-oxidation. Whether dietary OPA remains intact or is rapidly redistributed and metabolized in animals represents a key area for future research.

## 4. Sources and Distribution of OPA

### 4.1. Marine Microalgae as Primary Producers

OPA was first reported in microalgae in 1975 [47] and is now recognized as being produced predominantly by marine microalgae and phytoplankton [8,9,48,49]. Within microalgae, OPA is preferentially esterified to thylakoid membrane glycolipids, particularly monogalactosyldiacylglycerol (MGDG) and digalactosyldiacylglycerol (DGDG). Electrospray ionization mass spectrometry (ESI-MS/MS) reveals distinct regiochemical preferences; in many dinoflagellates and raphidophytes, OPA is localized predominantly to thylakoid galactolipids (MGDG and DGDG) at the *sn*-2 position paired with C20 PUFAs at the *sn*-1 position (e.g., 20:5/18:5), though symmetric di-OPA species (18:5/18:5) are also observed in glycolipids in some microalgae [37,38,50,51,52].

### 4.2. Relative Abundance Among Species

In most microalgal taxa, OPA constitutes only a minor fraction of total fatty acids, particularly relative to dominant VLCPUFAs such as EPA and DHA [49,53]. However, several documented dinoflagellate species accumulate notable levels of OPA, including *Prorocentrum micans, Gynodinium simplex, Symbiodinium microadriaticum* and *Scrippsiella trochoidea* [8,47]. Reported concentrations of OPA vary widely across taxa; for example, OPA accounts for approximately 15–18% and 20% of total fatty acids in *S. microadriaticum* and *S. trochoidea*, respectively, while in dinoflagellates species such as *Pyrocystis lunula* and *Protoceratium reticulatum*, OPA remains below 5% [48]. Remarkably, in dinoflagellates such as *Prorocentrium mexicanum* and *Gyrodinium aureolum*, OPA can account for up to 38% of total fatty acids [54,55,56]. Beyond Dinophyceae, OPA has also been found across Prymnesiophyceae and Raphidophyceae. Among prymnesiophytes, the coccolithophore *Emiliania huxleyi* is widely recognized as a major producer, with OPA levels ranging from 13.7% to 22.9% of total fatty acids across eight strains during the stationary growth phase. In contrast, the raphidophyte *Heterosigma akashiwo* exhibits lower OPA concentrations than *E. huxleyi* (Table 2) [37,57].

### 4.3. Environmental and Physiological Effects on OPA Accumulation

OPA accumulation in microalgae is highly selective and environmentally plastic. Unlike ALA, which is widely distributed among freshwater microalgae and terrestrial plants, OPA is almost exclusively produced by marine photosynthetic microalgae [7]. This restricted distribution indicates that the enzymatic machinery responsible for OPA biosynthesis is confined to specific marine algal lineages rather than being broadly conserved across photosynthetic taxa [48]. Cellular OPA content dynamically shifts in response to physiological and environmental conditions, including temperature, light intensity, salinity, nutrient availability and growth phase. For instance, in *Emiliania huxleyi*, reducing the growth temperature from 25 °C to 1 °C increased the OPA content from 24% to 27% of total fatty acids, accompanied by a 1.6-fold increase in transcription of the plastidial ω-3 desaturase gene *EhN3* [58]. Similarly, in *Heterosigma akashiwo*, OPA abundance reaches a maximum during the logarithmic growth phase before declining in the stationary phase [37]. These observations imply that OPA accumulation is environmentally and physiologically responsive, contributing to variation in its abundance across marine microalgal species and growth conditions.

## 5. Transfer Through the Marine Food Web

Although synthesized predominantly by marine microalgae, OPA enters the marine food web effectively when the phytoplankton are grazed upon by primary consumers, including zooplankton, copepods and krill [46,59]. The trophic transfer can extend to higher levels, including fish and invertebrates that feed directly on flagellates or zooplankton, thereby linking these organisms to OPA-producing phytoplankton communities [46,60]. However, unlike long-chain omega-3 fatty acids such as EPA and DHA, OPA does not accumulate to high concentrations in higher-trophic-level consumers. Instead, ingested OPA often undergoes rapid metabolic conversion rather than long-term storage [17,46].

## 6. Biological Function of OPA

While OPA is relatively low in abundance compared with major ω-3 LCPUFAs such as EPA and DHA in marine food webs, its distinctive structural properties and unique distribution among primary producers raise questions regarding its physiological and ecological functions in ecosystems. Current evidence suggests that the biological significance of OPA is primarily associated with its incorporation into photosynthetic membranes in response to environmental conditions, and with its potential contribution to nutritional and signaling processes across trophic levels (Table 3).

### 6.1. Biological Roles of OPA in Primary Producers

In marine primary producers, OPA is predominantly esterified within plastidial thylakoid membrane galactolipids, specifically MGDG and DGDG. Thus, a main physiological role of OPA is probably to maintain thylakoid membrane architecture, stability, and fluidity, thereby supporting photosynthetic efficiency and photoprotection under dynamic environmental conditions [10]. The hydrophobic core of a lipid bilayer must remain sufficiently fluid to allow the lateral diffusion of lipids and integral membrane protein complexes, including photosystems I and II, which is essential for electron transport and other membrane-associated activities [67]. This adaptive response, known as homeoviscous adaptation, involves dynamic changes in membrane lipid composition, including alterations in galactolipid headgroup ratios, acyl chain lengths, and degree of unsaturation in response to environmental conditions [67,68].

Phytoplankton routinely increase their fatty acid unsaturation in response to cold or nutrient stress. For example, in *Scenedesmus obliquus*, increasing growth temperatures decreased ω-3 PUFA abundance while increasing saturated fatty acids, indicating temperature-dependent remodeling of membrane lipids [69]. In this context, highly unsaturated fatty acids like OPA may provide structural advantages, as the five *cis* double bonds introduce the pronounced conformational kinks along the 18-carbon acyl chain, reducing acyl-chain packing and favoring membrane fluidity at low temperature. This physiological role is supported by many experimental observations. For instance, Okuyama et al. (1992) documented a significant increase in OPA when the growth temperature of a cold stenothermic Prymnesiophyte was reduced from 5 °C to 2 °C [11]. Similarly, cold stress in the picoalga *Ostreococcus tauri* induces the rapid transcriptional upregulation of a plastidial ω-3 desaturase accompanied by increased OPA accumulation in plastidial lipids [70]. Furthermore, structural lipidomic profiling of dinoflagellates has shown that cold exposure enriches OPA-containing MGDG and DGDG molecular species, particularly those containing OPA at the *sn*-2 position, consistent with a role in maintaining thylakoid membrane fluidity and photosynthetic function [38]. Together, these observations suggest that OPA accumulation and incorporation into membrane galactolipids are closely associated with environmental acclimation in microalga. This strategy may be particularly important for microalgae that predominantly synthesize OPA but produce little to no EPA and DHA. Since these species have restricted elongation and desaturation capacities, OPA may provide an alternative strategy to maintain thylakoid membrane properties under cold temperature conditions without relying on EPA and DHA.

Beyond their structural role in membranes, high unsaturated fatty acids can serve as substrates for the synthesis of oxylipins, a diverse group of oxygenated fatty acids involved in cellular signaling for stress responses [71,72]. In microalgae, environmental stresses such as nutrient limitation, changes in light, temperature and salinity can alter the production of oxylipins and other oxidized PUFA derivatives [73,74]. For example, Ulloa et al. (2025) demonstrated that silicon limitation significantly increases the extracellular release of oxygenated C18 polyunsaturated fatty acids, including OPA-derived oxylipins, during the logarithmic growth phase in diatoms such as *Pseudo-nitzschia multiseries*, indicating that OPA oxidation may contribute to early physiological stress responses and chemical signaling [61]. Similarly, Botana et al. (2026) demonstrated that OPA serves as a primary substrate for 13S-lipoxygenase in dinoflagellate microalgae, generating 13(S)-hydroxy-octadecapentaenoic acid [13(S)-HOPE], a novel oxylipin whose abundance increased sixfold under thermal stress, suggesting roles in maintaining cellular homeostasis and mitigating physiological stress [62]. Together, these findings provide direct evidence that OPA can serve as a substrate for oxylipin biosynthesis and may contribute to stress-responsive lipid signaling in microalgae. However, the exact physiological functions and signaling mechanisms of OPA-derived oxylipins remain to be explored.

### 6.2. Biological Roles of OPA in Consumers

OPA can enter marine food webs through the consumption of OPA-producing phytoplankton by zooplankton and other primary consumers. Zooplankton and invertebrates acquire PUFAs from their diet to support growth, reproduction, development and membrane function because many species have limited capacity to synthesize essential PUFAs de novo [60,63]. Environmental changes can alter phytoplankton lipid composition, which in turn affects the nutritional quality of their food and the physiological performance of consumers [75]. For example, the growth of *Daphnia*, a planktonic crustacean, is reduced under PUFA-poor conditions, highlighting the importance of dietary PUFAs for consumer fitness [64]. In this context, OPA-containing algal biomass may contribute to the dietary PUFA pool available to consumers and potentially influence consumer fitness.

Once consumed, OPA may be incorporated into cellular glycerolipids, converted to other fatty acids, or degraded through β-oxidation. If incorporated into consumer tissue glycerolipids, OPA may contribute to membrane structure and function through its high degree of unsaturation. Highly unsaturated fatty acids are important components of animal membrane lipids and contribute to the maintenance of membrane fluidity under changing environmental conditions. For example, fish modify the PUFA composition of brain and muscle membranes in response to temperature, with colder conditions generally favoring greater fatty acid unsaturation to preserve membrane function [65].

OPA may also contribute to the formation of bioactive lipid mediators following its entry into animal lipid pools. Oxylipins, such as eicosanoids derived from PUFA substrates, regulate diverse physiological processes such as immune and inflammatory responses, vascular and smooth muscle function, reproduction and growth [23,24]. However, direct evidence that OPA serves as a precursor for specific eicosanoids or other bioactive lipid mediators in consumers remains limited.

In addition to these potential physiological functions, OPA-containing algal materials may also be associated with adverse biological effects under certain conditions. Studies of dinoflagellate *Gymnodinium mikimotoi* have reported morphological damage to fish gills and intestinal tissues, as well as the disruption of cellular processes such as pH regulation and potassium uptake, in response to OPA-containing algal material [55,76]. These observations suggest that OPA or OPA-associated compounds may exert adverse effects at sufficiently high exposure levels, particularly during harmful algal blooms. However, the extent to which these effects are specifically attributable to OPA remains to be established.

### 6.3. Ecological Roles of OPA

At the ecosystem level, the significance of OPA may extend beyond its physiological roles in individual organisms to trophic transfer, release into dissolved organic matter (DOM), potential bioactive signaling, and its utility as an ecological biomarker. First, as discussed above, OPA-containing phytoplankton contribute to the PUFA pool available to consumers, and a decrease in the production of these PUFA-rich lipids may reduce prey quality and zooplankton fitness, with potential consequences for fish that depend on zooplankton as a food source and for energy transfer to higher trophic levels [60,77]. Reduced prey quality and quantity may therefore propagate through the food web, affecting higher trophic levels and the transfer of energy and essential lipids. Second, OPA can enter the dissolved organic matter (DOM) pool following the release and degradation of algal biomass. Cell lysis, senescence, grazing and viral infections can release membrane-derived fatty acids and other organic compounds into surrounding waters where they become available for microbial utilization and further transformation. Because OPA is predominantly associated with plastidial membrane lipids, its release may increase during periods of algal senescence, stress or mortality. Once released, OPA may serve as a carbon and energy source for heterotrophic microorganisms or undergo further microbial transformation, thereby contributing to the cycling of organic carbon and lipids in marine ecosystems [12,13,14]. Third, OPA and its oxidation products may contribute to chemical interactions among marine organisms. As discussed above, OPA can serve as a substrate for the formation of oxygenated lipid metabolites with potential biological activities. In the marine ecosystem, PUFA-derived oxidation products can also mediate interactions between phytoplankton and their consumers. For example, polyunsaturated aldehydes derived from diatom PUFAs have been shown to reduce copepod egg viability and recruitment, potentially influencing grazer population dynamics and predator–prey interactions [78]. Although similar ecological effects have not been demonstrated for OPA-derived metabolites, the formation of oxidized OPA derivatives under environmental stress suggests a potential role in chemically mediated interactions within marine food webs. Furthermore, the restricted distribution of OPA among marine primary producers may provide potential value as a biochemical biomarker. Unlike ubiquitous fatty acids such as palmitic acid, oleic acid, linoleic acid and ALA, OPA is uniquely enriched in certain marine microalgal groups, although its abundance can vary under environmental conditions. This distinctive distribution may therefore provide useful information for tracing the contribution of specific phytoplankton groups to marine food webs or identifying changes in phytoplankton community composition. For example, Imbs and Dembitsky (2023) identified OPA as a diagnostic lipid marker for symbiotic and free-living dinoflagellates; with its enrichment in thylakoid galactolipids, OPA can be used to distinguish dinoflagellate-derived organic matter from other microalgal inputs in marine environments [66]. However, the extent to which OPA can serve as a reliable ecological or taxonomic biomarker remains to be established, particularly given its potential metabolic modification and redistribution after transfer between trophic levels.

### 6.4. Potential Applications of OPA

Although research on the biological functions and applications of OPA remains limited, its occurrence as a marine-derived ω-3 PUFA and its structural and metabolic relationships with better characterized ω-3 PUFAs, particularly EPA and DHA, suggest several potential applications. In contrast to EPA and DHA, whose nutritional and physiological functions are well-established, evidence for OPA-specific benefits remains scarce [2,79]. Therefore, the applications discussed below are largely prospective and are based on the known biological properties of OPA, its metabolic relationship with other ω-3 PUFAs, and the established applications of structurally related marine lipids. Further investigation of OPA bioavailability, metabolism, safety, and biological activity will be necessary to determine whether these proposed applications can be translated into practical products.

### 6.5. Aquaculture and Animal Nutrition

Aquaculture and terrestrial animal feeding represent the most immediate and practical applications for OPA-rich marine microalgal biomass. Dietary ω-3 PUFAs are important for growth, development, and membrane function in farmed aquatic animals, particularly during early developmental stages [67]. Traditionally, these fatty acids are supplied through fish oils and fish meals; however, concerns regarding resource sustainability and supply have increased interest in microalgae as alternative sources of marine ω-3 PUFAs [53,80]. Several marine microalgae, including dinoflagellates and prymnesiophytes, naturally accumulate a high amount of OPA, making OPA-rich algal biomass a potential source of ω-3 PUFAs for aquaculture feed and animal feed.

The established nutritional importance of EPA and DHA provides a strong rationale for exploring the contribution of OPA-containing biomass to animal feeds. In aquaculture, algal diets enriched in ω-3 PUFAs increase lipid accumulation in tissues and gonads, directly enhancing egg quality, hatching success and reproductive performance in scallops such as *Pecten maximus* [53]. Similarly, microalgae are increasingly explored as sustainable sources of essential fatty acids for live feeds such as copepods, rotifers and brine shrimp [81]. OPA-rich microalgae could therefore be considered as alternative or complementary sources of dietary ω-3 PUFAs in aquaculture, potentially enhancing the lipid content and ω-3 PUFA availability of feeds and supporting membrane function, stress resistance and larval survival.

Beyond aquaculture, dietary microalgal PUFAs have also demonstrated significant utility in terrestrial livestock and poultry nutrition. In laying hens, dietary supplementation with microalgal biomass facilitates the efficient transfer of ω-3 fatty acids into egg yolk lipids, producing enriched table eggs with elevated EPA and DHA and a reduced n-6/n-3 ratio [82,83,84].

Nevertheless, direct feeding studies specifically evaluating purified OPA in aquaculture and animal diets remain scarce. It is currently unresolved whether OPA confers unique biological benefits beyond those contributed by the general PUFA profile of microalgal biomass. Future studies should prioritize determining OPA digestibility, intestinal absorption, tissue-specific accumulation and direct physiological effects on growth, reproductive and immune competence across target animal species.

### 6.6. Human Health and Nutraceutical Potential

Beyond its prospective role in aquafeeds and animal nutrition, OPA represents a promising candidate for direct human health applications and functional food development. Omega-3 VLCPUFAs, particularly EPA and DHA are clinically recognized for their cardioprotective, neuroprotective and anti-inflammatory properties. DHA is highly enriched in the brain and retina, where it supports neuronal and visual function, while serving as a substrate for specialized pro-resolving mediators, including resolvins and neuroprotectin D1 [3,4,20,21,85]. EPA contributes to inflammatory and cardiovascular regulation through its conversion to bioactive eicosanoids that modulate vascular function, immune responses and blood coagulation [18,19,74]. While OPA differs structurally by an additional double bond at the Δ3 position, its close metabolic relationship as an upstream precursor to EPA suggests that it is a compelling candidate for future functional and pharmacological studies (Figure 3). In addition, it is possible that OPA-derived oxylipins could also function as signaling molecules. However, the biological activity of these metabolites in human systems remains unexplored. Furthermore, ω-3 fatty acids have been linked to neural development and metabolic regulation; as such, OPA may also provide similar benefits [3,86]. As the demand for ω-3 PUFAs continues to grow, OPA-enriched supplements could become a novel effective ingredient in health and nutrition, although their safety, bioavailability, and beneficial effects remain to be established.

### 6.7. Other Applications

The potential applications of OPA can extend beyond aquaculture and human nutrition into other fields such as pharmaceuticals, cosmetics and environmental science. Marine microalgae rich in ω-3 PUFAs are increasingly being investigated for topical applications. For instance, microalgal lipids are incorporated into cosmetic formulations for their skin protective properties, which enhance skin elasticity, improve smoothness, and provide UV protection [87,88]. Given its potential anti-inflammatory and skin-protective profile, OPA could serve as an active ingredient in formulations for sensitive skin and calming lotions. Although specific studies on OPA remain limited, structurally similar PUFAs are already being explored for these cosmetic and pharmaceutical areas [89,90,91].

In ecology, the unique presence of OPA in certain microalgal species positions it as a promising trophic biomarker [59]. Since fatty acids from primary producers are conservatively transferred through food webs, OPA could serve as a tracer to identify predator–prey relationships and elucidate feeding strategies in marine ecosystems [46,92]. Finally, in the context of sustainable energy where microalgae biomass has been utilized as a feedstock in biofuel production, extracting OPA as a co-product could contribute significant value to this production system [53,93,94]. These diverse applications highlight the versatility of OPA, though realizing its full potential will depend on future research to understand its physiological functions and practical feasibility (Table 4).

## 7. Challenges and Future Directions

### 7.1. Limited Understanding of Biological Functions

One of the major gaps in OPA research is the limited understanding of its biological functions in primary producers and consumers. While some evidence suggests that OPA contributes to membrane fluidity in certain microalgae, direct experimental studies examining its specific functional role remain scarce. Most current knowledge is inferred from its structural similarity to EPA and DHA, or from general PUFA dynamics. In primary producers, OPA is localized predominantly in plastidial membrane lipids, suggesting a structural role in photosynthetic membranes [34,40]. However, it remains uncertain whether OPA confers other physiological advantages under environmental stress, acts as a critical precursor for stress-signaling oxylipins, or simply functions as a metabolic intermediate.

In consumers, available evidence suggests that OPA is rapidly metabolized, but critical questions remain regarding the extent to which OPA is incorporated into specific glycerolipids, elongated to longer chain PUFAs, or further metabolized. Since most dietary studies prioritize EPA and DHA, OPA’s contribution to physiological performance, immune competence, and reproductive fitness remains understudied, leaving it largely overlooked in nutritional application. In humans, the physiological role of OPA is almost entirely unexplored. Controlled clinical or nutritional trials are needed to determine its metabolic fate, safety and bioactivity. Until such targeted human or animal studies are conducted, it remains unclear whether OPA offers unique health benefits or functions primarily as a precursor for the biosynthesis of ω-3 VLCPUFAs.

### 7.2. Barriers to Production

Despite its prospective health benefits, OPA is not readily available from abundant natural sources, making scalable production a critical bottleneck. The natural abundance of OPA is generally low and highly variable, restricted primarily to the membrane lipids of selected marine microalgae [7,8]. This natural limitation poses a considerable barrier to large-scale production for aquaculture and nutraceuticals markets. Unlike EPA and DHA that are established commercial commodities derived from heterotrophic microalgal fermentation or fish oil, OPA is not yet amenable to high-yield enrichment or downstream recovery. In native producers, cultivating wild-type strains under abiotic stressors, such as nutrient depletion, low temperature, salinity shifts, or UV radiation, represents one way to stimulate overall lipid accumulation and maximize cellular OPA yields [53,95]. However, wild-type OPA-accumulating microalgae have not undergone systematic bioprocess optimization, and the harvest yields of OPA remain unknown. As such, metabolic engineering of OPA in heterologous systems offers an attractive alternative, though it presents several biochemical challenges. First, the underlying biosynthetic pathway and associated enzymes must be elucidated and identified before effective production can be implemented. A recent tracer study in the microalga *E. huxleyi* using ^14^C-labeled acetate demonstrated that OPA is synthesized plastidially within galactolipids, such as MGDG and DGDG through sequential desaturation with the last step being a Δ3 desaturation [34]. However, to date, no front-end PUFA Δ3 desaturase has ever been identified, and the gene encoding the enzyme has not yet been cloned in any living organism. Therefore, one of the primary future efforts is to identify and characterize the gene for the biosynthesis of OPA in microalgae. Second, because OPA accumulates mainly in galactolipids rather than storage lipid triacylglycerols (TAGs), producing a high level of OPA in heterologous systems will require channeling it into TAGs effectively. Thus, another key future research direction is to identify specific acyltransferases such as diacylglycerol acyltransferases (DGATs) and phospholipid:diacylglycerol acyltransferases (PDATs) that can effectively acylate OPA into the glycerol backbone to yield triacylglycerols. A combination of these biosynthetic and assembling enzymes will be required for the metabolic engineering of OPA in heterologous systems.

### 7.3. Analytical Challenges

To a lesser extent, progress in OPA research is held back by analytical issues associated with its detection, identification and quantification. A major issue with standard non-polar gas chromatography (GC) columns (such as Equity-5) is co-elution, where OPA’s retention time can overlap with C20:1 fatty acid isomers (e.g., 20:1n-7 and 20:1n-9) or other abundant PUFAs, whereas polar capillary columns (such as DB-23) successfully resolve the OPA peak [7,34]. As noted in early work by Napolitano et al. (1988), this co-elution makes it difficult to accurately identify chromatographic peaks, potentially causing OPA to be missed or underreported in marine lipid profiles [96]. In addition, chemical instability during sample preparation might further complicate OPA analysis [97]. During derivatization for GC and GC-MS analysis, Svetashev & Imbs (2014) showed that OPA can undergo isomerization, creating additional isomers that complicate accurate peak identification and quantification [98]. These analytical challenges, combined with OPA’s low natural abundance, make it particularly susceptible to being overlooked in complex lipid extracts or environmental samples. Therefore, standardized and validated analytical protocols are needed to accurately identify and quantify OPA. Encouragingly, significant progress has been made in improving analytical methods for PUFAs. Beyond gas chromatography, several other analytical approaches have been developed to improve the separation and identification of these fatty acids and their lipid derivatives, including GC-MS and ESI-MS/MS (electrospray ionization tandem mass spectrometry) for determining the identity and regiospecific distribution in different lipid classes [42,98].

### 7.4. Concluding Remarks

Octadecapentaenoic acid is an emerging yet frequently overlooked member of the ω-3 PUFA family. While structurally related to well-characterized ω-3 VLCPUFAs such as EPA and DHA, OPA is distinguished by its shorter carbon chain and high degree of unsaturation. These unique structural features largely dictate its physicochemical behavior, metabolic fate and functional roles. Current evidence indicates that OPA is synthesized exclusively by certain marine microalgae, where its proposed functions are to maintain plastidial membrane integrity for photosynthesis and fluidity under environmental stress, and potentially serves as a metabolic precursor for long chain PUFAs or stress-signaling bioactive oxylipins. Unlike EPA and DHA, which are conservatively transferred and accumulated across higher trophic levels, OPA is rapidly metabolized by consumers, acting primarily as a metabolic intermediate or an oxidizable energy source rather than a stored lipid. The potential applications of OPA in aquaculture and animal nutrition are promising, but their realization is currently hindered by significant production bottlenecks. There is no abundant natural source of OPA, and efforts to establish heterologous production platforms through metabolic engineering are limited by the fact that the gene encoding the critical Δ3 PUFA desaturase remains unidentified. Furthermore, the understanding of OPA’s physiological roles in humans relies heavily on structural related established ω-3 fatty acids rather than direct experimental evidence. Consequently, OPA remains a fatty acid of considerable interest but with many unanswered questions regarding its metabolic destiny and specific therapeutic benefits. Future research must prioritize identifying the elusive Δ3 desaturase gene to enable scalable production through metabolic engineering, alongside targeted in vivo studies to determine whether OPA’s distinct structure confers unique biological advantages. Ultimately, clarifying these functional properties and metabolic pathways will be essential to establish OPA’s commercial viability and justify its development as a novel functional ingredient or dietary supplement.

## Figures and Tables

**Table 1:** Structural and functional comparison of OPA with related polyunsaturated fatty acids.

Fatty Acid	Abbreviation (Notation)	Chain Length	Double Bonds	Omega Class	Primary Known Function	References
Octadecapentaenoic acid	OPA (18:5n-3)	18	5	ω-3	Potential precursor, membrane structure, biomarker.	[10,11,12,13,14]
α-Linolenic acid	ALA (18:3n-3)	18	3	ω-3	Essential fatty acid, membrane structure, precursor to EPA/DHA.	[15]
Stearidonic acid	SDA (18:4n-3)	18	4	ω-3	Membrane structure, precursor to EPA and DHA, biomarker.	[16,17]
Eicosapentaenoic acid	EPA (20:5n-3)	20	5	ω-3	Precursor to anti-inflammatory eicosanoids, cardiovascular function, immune activity.	[6,18,19]
Docosahexaenoic acid	DHA (22:6n-3)	22	6	ω-3	Membrane structure, precursor to docosanoids, anti-inflammation, neuroprotection, immune activity.	[3,4,20,21]
Arachidonic acid	ARA (20:4n-6)	20	4	ω-6	Precursor to pro-inflammatory eicosanoids, cell signaling, membrane structure.	[22,23,24]

**Table 2:** Occurrence and relative abundance of OPA (% of total fatty acid) in marine microalgae.

Species	Taxonomic Class	OPA (%)	Source
*Peridinium triquetrum*	Dinophyceae	22.6	[47]
*Scrippsiella trochoidea*	Dinophyceae	20	[48]
*Gymnodinium breve*	Dinophyceae	19.4	[47]
*Symbiodinium microadriaticum*	Dinophyceae	15–18	[48]
*Emiliania huxleyi*	Prymnesiophyceae	13.7–22.9	[57]
*Prorocentrum micans*	Dinophyceae	12.5–34	[47,48]
*Prorocentrum mexicanum*	Dinophyceae	37.6	[50]
*Gymnodinium breve*	Dinophyceae	11.9	[47]
*Protoceratium reticulatum*	Dinophyceae	5	[48]
*Pyrocystis lunula*	Dinophyceae	3	[48]
*Heterosigma akashiwo*	Raphidophyceae	<5	[37]

**Table 3:** Potential biological functions of OPA.

Biological Level	Potential Functions	Supporting Evidence	Key References
Cellular (primary producers)	Membrane adaptation: Contributes to membrane fluidity under cold stress	General role of PUFAs in cold adaptation	[10]
		Increased OPA in prymnesiophyte membranes at 2 °C vs. 5 °C	[11]
	Metabolic intermediate: Potential precursor for ω-3 VLCPUFA biosynthesis	Substrate for further elongation and desaturation	[7]
	Oxylipin precursor: Contributes to stress-related lipid signaling	OPA-derived oxylipin increases under silicon limitation	[61]
		OPA-derived 13(S)-HOPE increases under thermal stress	[62]
Physiological (consumers)	Nutritional value: Contributes to the dietary PUFA pool	OPA-containing algal biomass may contribute to the dietary PUFA pool available to consumers	[49,63,64]
	Metabolic flexibility: Can be converted or oxidized for energy	Rapid OPA conversion in fish cell cultures; limited accumulation at higher trophic levels	[17]
	Membrane and signaling roles: Membrane function and bioactive lipid mediator production	Similarity to PUFAs involved in membrane function and lipid signaling	[24,65]
	Adverse effects: Cellular and tissue damage at high exposure	OPA-containing *Gymnodinium mikimotoi* materials associated with gill and intestinal damage in fish	[55]
Ecological	Contribution to dissolved organic matter: Enters organic matter pools following algal degradation	OPA can be released during cell lysis, senescence, grazing or viral infection and undergo microbial transformation	[12,13,14]
	Ecological biomarker: Traces contributions from specific phytoplankton groups	OPA enrichment distinguishes dinoflagellate-derived organic matter from other microalgal inputs	[66]

**Table 4:** Potential applications, supporting evidence and key research gaps for OPA across diverse sectors.

Sector	Potential Applications	Supporting Evidence	Research Needed
Aquaculture	Feed enrichment: Improve nutritional value of live feeds (e.g., rotifers, copepods, shrimp)	Known roles of ω-3 VLCPUFAs in larval growth, development, and survival	Feeding trials evaluating direct uptake, survival rates, and physiological performance in target species
		Structural & functional similarity to EPA/DHA	
Human health & nutrition	Novel ω-3 nutraceutical: Anti-inflammatory, cardiovascular, and immune function support	Established roles of ω-3 PUFAs as oxylipin precursors	Clinical and in vitro trials on human bioavailability, bioactivity, safety and therapeutic mechanisms
		Potential bioactivity of OPA-derived signaling mediators	
Other Emerging Uses	Cosmetic & dermatology: Topical skin barrier repair, anti-inflammatory, and UV-protective formulations	Demonstrated efficacy of marine ω-3 PUFAs in improving skin elasticity and soothing inflammation	In vitro and in vivo dermatological assays testing OPA stability, skin penetration, and bioactivity
	Ecological biomarker: Trophic tracer for food web dynamics and predator-prey interactions	Unique presence of OPA in specific microalgal taxa; conservative fatty acid trophic transfer across trophic levels	Validation of OPA trophic transfer efficiency and metabolic retention in marine food webs

## Data Availability

No new data were created or analyzed in this study. Data sharing is not applicable to this article.

## Abbreviations

ALA, α-linolenic acid; ARA, arachidonic acid; CoA, coenzyme A; DAG, diacylglycerol; DGDG, digalactosyldiacylglycerol; DGAT, diacylglycerol acyltransferase; DHA, docosahexaenoic acid; DOM, dissolved organic matter; DPA, docosapentaenoic acid; EPA, eicosapentaenoic acid; ESI-MS/MS, electrospray ionization tandem mass spectrometry; ETA, eicosatetraenoic acid; FAS, fatty acid synthase; GC, gas chromatography; GC-MS, gas chromatography-mass spectrometry; HOPE, hydroxy-octadecapentaenoic acid; LA, linoleic acid; MGDG, monogalactosyldiacylglycerol; OA, oleic acid; OPA, octadecapentaenoic acid; PC, phosphatidylcholine; PDAT, phospholipid:diacylglycerol acyltransferase; PG, phosphatidylglycerol; PUFA, polyunsaturated fatty acid; SDA, stearidonic acid; SQDG, sulfoquinovosyldiacylglycerol; TAG, triacylglycerol; UV, ultraviolet; VLCPUFA, very-long-chain polyunsaturated fatty acid.
